# The EMPA-REG outcome study: critical appraisal and potential clinical implications

**DOI:** 10.1186/s12933-016-0403-8

**Published:** 2016-06-04

**Authors:** Gianluca Perseghin, Anna Solini

**Affiliations:** Metabolic Medicine, Policlinico di Monza & Department of Biomedical Sciences for Health, University of Milan, Via Amati 111, 20900 Monza, Italy; Department of Clinical and Experimental Medicine, University of Pisa, 56126 Pisa, Italy

**Keywords:** Cardiovascular safety, Hypoglycemic agents, Type 2 diabetes, Empagliflozin

## Abstract

Diabetes health care professionals have to face a study with results of incomparable success in secondary and tertiary cardiovascular disease prevention. In the past, no studies in patients with type 2 diabetes resulted to be successful in inducing an improvement of cardiovascular prognosis, no matter whether they were focused on a target, on life-style or on pharmacological intervention. On a clinical perspective, should the diabetologist’s way to think about the anti-diabetic therapy of patients on secondary cardiovascular prevention change based on the results of *Empa*-*Reg outcome?* Due to the complexity of the clinical picture of patients with type 2 diabetes, a tailored therapy based on targets, complications, co-morbidity, familial and social environment, personal and cultural features must be conceived and applied in starting pharmacological therapy; however, the question whether should we consider empagliflozin as first choice therapy in individuals with type 2 diabetes exposed to high cardiovascular risk, the *Empa*-*Reg outcome*-like patient, awaits now for an answer. Waiting for data confirming the results of the *Empa*-*Reg outcome study,* this report goes through the good reasons in support of this way of thinking, but at the same time explores the many unanswered questions raising potential concerns about this clinical choice.

## Brief summary of the study

### Aim and patients

- *Aim*: To test the non-inferiority of empagliflozin vs placebo in terms of cardiovascular (CV) safety.
- *Patients*: 7020 type 2 diabetes (T2DM) individuals with established CV disease (defined as the presence of ≥1 of the following: history of myocardial infarction; documented multi-vessel coronary artery disease; documented single-vessel coronary artery disease with ≥50 % luminal narrowing plus positive non-invasive stress test for ischemia or recent hospital discharge for unstable angina; documented unstable angina; history of ischemic or hemorrhagic stroke; occlusive peripheral artery disease), randomized to placebo, empagliflozin 10 or 25 mg as add-on to the usual therapy.
- Patients allocated in the three arms were comparable for age, gender, BMI and HbA1c; blood pressure (BP) and LDL-cholesterol levels (mean 135/77 mmHg and 85–86 mg/dl, respectively) were in a reasonably good control; 95 % of them assumed antihypertensive drugs, 80 % hypolipidemic drugs and 83 % anti-platelets drugs.
- *Median treatment duration*: 2.6 years; median observation time 3.1 years.
- *Statistical analysis*: intention-to-treat, comparing response to empagliflozin 10 and 25 mg vs placebo in all subjects receiving at least one treatment dose; non-inferiority analysis, with a HR border of 1.3 vs placebo for primary and secondary endpoint; superiority was tested as eventual following step.

## Results

- *HbA1c* At the end of the study HbA1c reduction was 0.24 % with 10 mg and 0.36 % with 25 mg empagliflozin vs placebo.
- *Body weight* Patients randomized to empagliflozin showed a significant reduction in body weight (2–3 kg with empagliflozin 25 mg).
- *Arterial BP and heart rate* Empagliflozin induced a prompt reduction of systolic BP (4–6 mmHg vs placebo at week 16), which was maintained along the time. At the end of the study diastolic BP did not differ from placebo. A higher percent of placebo-treated patients required a potentiation of the background anti-hypertensive therapy. No difference in the heart rate was observed.
- *Lipid profile and uric acid* Active treatment induced an initial raise in LDL-cholesterol (3–4 mg/dl vs placebo), which resulted to be negligible after 52 weeks. HDL-cholesterol showed a similar trend in the three arms. The uricosuric effect of empagliflozin was confirmed.

The main results of the study in terms of CV endpoints are shown in Table 1. The effects on hard endpoints did not differ in the two empagliflozin arms.

**Table 1:** Cardiovascular results of the *Empa-Reg* outcome study

	Empagliflozin (all together)	Placebo	HR 95 % CI	Non inferiority	Superiority
Primary endpoint	490/4687 (10.5 %)	282/2333 (12.1 %)	0.86 0.74–0.99	P < 0.001	P = 0.04
Key secondary endpoint	599/4687 (12.8 %)	333/2333 (14.3 %)	0.89 0.78–1.01	P < 0.001	P = 0.08
Cardiovascular death	172/4687 (3.7 %)	137/2333 (5.9 %)	0.62 0.49–0.77	N.A.	P < 0.001
Non-fatal myocardial infarction	213/4687 (4.5 %)	121/2333 (5.2 %)	0.87 0.70–1.09	N.A.	P = 0.22
Non-fatal stroke	150/4687 (3.2 %)	60/2333 (2.6 %)	1.24 0.92–1.67	N.A.	P = 0.16
Heart failure	126/4687 (2.7 %)	95/2333 (4.1 %)	0.65 0.50–0.85	N.A.	P = 0.002
All-cause death	269/4687 (5.7 %)	194/2333 (8.3 %)	0.68 0.57–0.82	N.A.	P = 0.001

Primary endpoint: standard 3 endpoint-MACE (CV death, non-fatal myocardial infarction (MI), non-fatal stroke); pre-specified key secondary endpoints: time to first event (CV death, non-fatal MI, non-fatal stroke, hospitalization for unstable angina)

*N.A.* not applicable

### *The Empa*-*Reg outcome* is a rewarding study with respect to other clinical trials comparing a specific anti-hyperglycemic drugs vs placebo in terms of CV endpoints

It is difficult to compare studies performed in different historical periods, with different aims, and in patients with different clinical characteristics and concomitant treatments; however, an attempt to compare *Empa*-*Reg outcome* [1] with recent clinical studies aimed at assessing non inferiority of other novel anti-diabetic drugs respect to traditional, established therapies, is imperative.

The *PROactivestudy* tested the efficacy of pioglitazone in reducing CV morbidity and mortality in high CV risk T2DM patients [2]. The primary endpoint (all-cause mortality, non-fatal MI, non-fatal stroke, acute coronary syndrome, revascularization procedures or lower limb amputation) was not achieved, in contrast with a matched secondary endpoint (CV death, MI, stroke). The study was characterized by an excess of non-fatal heart failure (HF) but it should be emphasized that HF was not an adjudicated end-point and that cases of HF resulted to have fewer CV events than those observed in the placebo group, raising doubt about the incidence of a true HF which could be misclassified in the place of peripheral edema. Moreover, the only endpoint with unfavorable outcome was the procedure of peripheral revascularization, in contrast with the effect on MACE which was consistently a positive one.

The *Origin study* tested the efficacy of glargine in reducing CV morbidity in T2DM patients with whatever background therapy, including insulin [3]. Characteristics of the participants were different from those of *Empa*-*Reg outcome,* being patients with altered glucose tolerance or recent onset diabetes even though approximately 60 % of them were on secondary CV prevention. The trial generated a neutral result in terms of coprimary endpoints (nonfatal MI, nonfatal stroke, or death from CV causes and these events plus revascularization or hospitalization for heart failure.), even if it should be underlined that the daily insulin use resulted to be about 30 IU per day, likely due to very short duration of the disease. Some patients, especially those with a longer duration, may require a significantly higher amount of insulin and in our opinion this condition remains to be tested in terms of CV safety.

*Savor*-*TIMI* [4], *Examine* [5] and *Tecos* [6] tested the effects of the DPP-IV inhibitors saxagliptin, alogliptin and sitagliptin on CV safety, to demonstrate their non-inferiority with respect to placebo, as requested by regulatory authorities. Recruited patients differed for clinical characteristics: high CV risk patients in *Savor*-*TIMI*; individuals with recent acute coronary syndrome in *Examine*; relatively low CV risk patients in *Tecos*. We interpret the general findings of these studies as a demonstration of DPP-IV inhibitors neutrality on CV risk, though a higher risk of hospitalization for HF was detected in *SAVOR*-*TIMI* and for some authors, based on retrospective calculations of the sample size, also in Examine. These correlative findings highlighted the potential relation between anti-hyperglycemic therapies and risk of HF, probably due to the link with the weight gain often associated with this treatment [7]. More recently at least three very large studies reported no effects of DPP-IV inhibitors when tested retrospectively in the clinical setting [8, 9] or very small effects [10].

Studies evaluating the effects of GLP-1 analogues will hopefully contribute to clarify this important issue. At this stage *Elixa* [11], performed in patients with recent acute coronary syndrome, documented the CV safety of lisixenatide when compared to placebo, and we are now waiting for the publication of the results of the *LEADER* trial, also testing CV safety of one of another GLP1-RA, liraglutide, which is expected in the next few weeks.

### EMPA REG outcome is a relevant success also with respect to trials comparing conventional vs intensive strategies of glucose control on CV endpoints

Since the *UKPDS study* has been published [12], the international community is still debating whether or not optimizing glucose control could reduce CV risk. To address this question, three main large randomized clinical trials were completed, aimed at verifying whether or not an improved metabolic control (irrespective of how this was obtained) could be associated with a better CV outcome: *ACCORD* [13], *ADVANCE* [14] and *VADT* [15]. It is well known that these studies have generated negative results and frustration, even if they resulted to be very useful in inducing a change in the vision of how we have to treat our patients, emphasizing the need for a “tailored therapy” able to address the individual needs of each single patient, especially with respect to the targets to pursue.

A common thinking is that an intensive anti-hyperglycemic therapy can be deleterious on mortality rate of frail individuals, therefore neutralizing potential CV benefits [13, 15]; however, it should be noticed that the prolonged observation of the individuals participating to two of those trials [16, 17] detected, in the post-trial observational period, that patients treated more intensively had a reduced CV risk which took longer than it was thought, confirming the previously reported and similar findings of the follow-up of the UKPDS study [18].

Also when combined physical exercise and nutritional therapy were the selected strategy to improve glucose control, as it was tested in the *Look Ahead* study, a CV benefit could not be detected [19]. Only the *PREDIMED* study [20], which tested the cardiovascular impact of the Mediterranean Diet, reported some positive results. It this study 7447 persons at high CV risk (with and without diabetes), but without CV disease at baseline were randomized to three different dietary regimens: standard traditional diet, Mediterranean diet enriched with olive oil or with nuts and almonds. Primary end-point was the classical composite cardiovascular death, non fatal MI and non fatal stroke. Even if the nutritional intervention had no effect on mortality, it showed a clear-cut positive effect on CV events which could be observed early during the observational period suggesting that the nutritionally-induced benefit could be mediated via and anti-inflammatory effect.

### The mechanisms by which empagliflozin determined the CV protection observed in the *Empa-Regoutcome* remain, so far, obscure

*Empa*-*Reg outcome* is a successful clinical study, opening novel scenarios but also leaving unsolved several questions. Mechanistically, it is unclear how the treatment could determine such CV benefit. Several candidates might be taken into consideration.

*Anti*-*hyperglycemic effect* in our opinion the hypothesis that the beneficial CV impact could be mediated via the anti-hyperglycemic effect is weak because the difference in HbA1c levels during the study between empagliflozin treatment and placebo was too small to explain the outcome. More importantly, the rapid onset of the protective effect of the treatment in the empagliflozin arms seems to be not compatible with a glycemic effect, which is supposed to take longer time as discussed above [16–18]; such effect has been explained with the concept of “metabolic memory”, which was detected years after the intervention, not in few weeks/months, as observed in *Empa*-*Reg outcome*. However empagliflozin has recently shown a fast (24 h) efficacy in reducing post-prandial glucose and improving metabolic control in Japanese T2DM individuals [21].

*Weight loss* similar explanations minimize the possibility that the positive effect could be related to the weight loss. Also in this case the small difference among the groups in terms of body weight change does not seem sufficient to explain such a large and rapid effect on CV mortality; however, we should not forget that, in these high CV risk patients, rapid weight gain, potentially reflecting fluid retention, can be considered a “proxy” for the risk to develop HF [7]. However, a recent pooled analysis performed on 3300 patients receiving empagliflozin has shown its ability to reduce waist, total body fat and indexes of central and visceral adiposity, likely contributing to reduce CV risk [22]. The sustained reduction of weight loss and HbA1c might have played a role in maintaining a lower CV risk along the whole duration of the trial [23].

*BP reduction* the significant drug-induced difference in systolic BP could potentially have strongly contributed to the results; this effect is also compatible with the early divergence of the survival curves, thus playing a major role in the improved prognosis of empagliflozin-treated patients. Against this hypothesis stands the observed trend toward an increased risk of non-fatal stroke; this is surprising because stroke is the macrovascular complication that should mostly take advantage from BP reduction.

*Uricosuric effect* recently, the role of uric acid as determinant of CV disease has been postulated [24], also in the light of an association between uric acid levels and HF [25]; some studies showed the beneficial effect of lowering uric acid on CV events [26, 27], even though data are still controversial [28] and mechanisms are still unknown. On this basis, the uricosuric effect of SGLT2 inhibitors could be a potential candidate to explain CV protection even if perplexity remains with respect to the rapid onset of protection that makes difficult to sustain this metabolic hypothesis.

*Inflammation* an interesting phenomenon observed in the trial is the early opening of the rate of events, that makes unlikely an effect on the natural history of atherosclerotic disease and its acute manifestations. The relatively low impact on MI and stroke seems to support this view. It should be noted that, up to date, the effect of SGLT2 inhibitors on systemic inflammation is unknown, even though data obtained in animal models show a reduced expression of tissue markers of inflammation and oxidative stress following SGLT2 inhibitors administration at the level of the kidneys [29] and, more recently, of the β-cell, with reduced apoptosis and tissue expression of reactive oxygen species [30]. It is interesting to note the parallelism with the results of the *PREDIMED* study, in which the early effect on CV protection was ascribed to an anti-inflammatory impact of the Mediterranean diet.

*Glucoretic effect* the beneficial CV effect could be explained by the reduction of the risk of heart failure and related cardiovascular risk condition, in particular sudden death [31]. This view is supported by the marked reduction (−35 %) of hospitalization for HF. If this is the case, the effect could be mediated by the reduction of plasma volume and cardiac pre-load driven by the glucoretic and natriuretic effect of the SGLT2 inhibitor, and is compatible with the early onset of the beneficial effect in the observational period. In our opinion this interpretation is clinically relevant because it further reminds to health care professional the prognostic importance of HF in patients with T2DM also considering the fact that not only overt but also subclinical HF is a frequent finding and/or suspicion in our patients in the outpatient setting.

*Anti*-*arrhythmic effect* another factor explaining the rapid occurrence of CV protection induced by empagliflozin could be an anti-arrhythmic effect, indirectly mediated by glucagon [32], whose release is increased by empagliflozin [33].

*Increased cardiac ß*-*hydroxybutyrate uptake* it may be hypothesized that under the above conditions of stimulated glucagon release, mild, persistent hyperketonemia, like may occur during treatment with SGLT2 inhibitors as reported by Ferrannini et al. [34], β-hydroxybutyrate may be freely taken up by the heart and oxidized in preference to fatty acids. This fuel selection may improve transduction of oxygen consumption into work efficiency at the mitochondrial level. In addition, the hemoconcentration that typically follows SGLT2 inhibition likely enhances oxygen release to the tissues, thereby establishing a powerful synergy with the metabolic substrate shift and finally explaining the beneficial cardiovascular effect observed in *Empa*-*Reg outcome.*

*RAAS*-*mediated effect* the vast majority of participants in the *Empa*-*Reg outcome* study were treated with RAAS active drugs. Combining the two regimens (RAAS blocking and SGLT2 inhibition), as already suggested to explain positive effects on the glomerular hemodynamics and nephroprotection [35], might in principle activate the AT2 receptor and the Angiotensin 1–7 pathway, with an anti-proliferative, anti-inflammatory, anti-arrhythmic, vasodilatory effect.

*Arterial stiffness* empagliflozin has been reported to reduce arterial stiffness in normotensive T1DM individuals [36] and could contribute to the beneficial CV effects. In *Empa*-*Reg outcome* this parameter was not measured, therefore its potential role is hypothetical and not supported by experimental data at this stage.

### Safety profile in the *Empa*-*Reg outcome* study

The *Empa*-*Reg outcome* study showed a surprising safety profile across the whole line of previously reported potential adverse events and side effects. The most insidious one, diabetic ketoacidosis, occurred rarely (0.1 %), without difference between active treatment and placebo. Similarly, elderly patients, apparently those with the lowest indication to use SGLT2 inhibitors, have taken advantage from the best prognosis, even though it will be important to better define the relationship between increased blood cells count and the small, not significant signal of risk of stroke. In parallel, patients with chronic kidney disease showed a documented prognostic benefit, similar to that of individuals with preserved renal function. Finally, the reported increased risk of genital tract infection was fully confirmed, though its impact on the need for drug withdrawal was small.

### Approaching other SGLT2-inhibitors studies

To answer the question whether or not this CV protection is a class effect, or is exclusive of empagliflozin is, at this stage, a difficult task. If we postulate that the beneficial cardiovascular effect is mostly related to glucoretic/natriuretic effect and consequent volume depletion with a parallel, additional reduction of systolic BP or if we consider that it may be mediated via metabolic effects related to increased cardiac ketone bodies uptake and disposal it is likely that benefit can be considered a class-effect because they might be all able to induce these effects. Said that, different drugs may have different specificity for SGLT2 and SGLT1, opening to discussion and speculation related to many other different potentially relevant effects in terms of CV protection making potentially different the effect of each single drug within the class. For this reason we believe that it is imperative to confirm the results of *Empa*-*Reg outcome*. In few years, results from *DECLARE* (dapagliflozin in patients with a lower CV risk patients) and *CANVAS* (canagliflozin in high CV risk individuals) studies will be available, confirming or denying the role of SGLT2 inhibitors as first-class anti-diabetic drugs able to manage residual CV risk up to reduce mortality, likely independently from their glucose-lowering effect.

### Which will be the impact on microvascular complications?

Forthcoming studies with other SGLT2 inhibitors should also address still open questions regarding the effect on microvascular complications. In fact, the anti-hyperglycemic effect of empagliflozin might still be unsatisfactory for many patients in a real-life context. In *Empa*-*Reg outcome* the HbA1c reduction of 0.3–0.4 % from baseline to the end of the trial failing to achieve the general target of <7 %, which is recognized to guarantee a robust protection against the risk to develop microvascular complication, would maintain concern with respect to this clinical issue. When considering kidney disease, *Empa*-*Reg renal* will likely answer in a while; promising preliminary data, showing a stable glomerular filtration rate across the whole study duration vs a decline in the placebo arm, were shown at the American Society of Nephrology 2015 annual meeting and we are waiting for the publication of the original article. This beneficial effect on diabetic kidney disease may be selectively explained by the capacity of empagliflozin to prevent hyperfiltration in the early stage of diabetic nephropathy as reported in patients with type 1 diabetes [37]. The question about microvascular complication would remain open with respect to diabetic retinopathy and neuropathy in which mechanistically the above mentioned protective mechanism for the kidney may result to be irrelevant for the eyes and the peripheral nervous system.

## Conclusion

Whatever would the future scenario be, *Empa*-*Reg outcome,* with the strength of its results, can likely influence the modality how, in the last years, we have considered the available therapeutic options. Compared with other clinical trials, we face for the first time an anti-diabetic drug able to reduce the risk of death, CV death and HF. Such results have no precedents, and the clinician cannot ignore it.

The paradigm has changed after this study. When we figure out an *Empa*-*Reg outcome*-like T2DM patient, before *Empa*-*Reg outcome* we would ask ourselves which could have been the best “tailored” therapeutic intervention, on the basis of his/her individual features (age, personal clinical history, diabetes duration, glycemic target, phenotype, lifestyle, presence of complications and/or comorbidities). Now, we should establish whether in *Empa*-*Reg*-like patients there is any reason, except the presence of precise contra-indications to its use, for not considering the possibility to prescribe a drug able to improve the prognosis of our patient irrespective of its anti-hyperglycemic effect. What has been observed during the *Empa*-*Reg outcome* study remains, so far, an enigma, and it seems difficult to use a therapeutic instrument without knowing the mechanism throughout it improves CV prognosis, especially when such protection does not seem to be mediated by atherosclerosis-related mechanisms.

We are in front of a single clinical trial, even robust and well-conducted. More data will be necessary to confirm these positive effects, perhaps providing instruments to understand what has exactly happened in *Empa*-*Reg outcome.*

However, such therapeutic approach should be seriously considered because, in *Empa*-*Reg outcome*, body weight, BP, uric acid levels, risk of hypoglycemia, all go in the desired direction. The study also confirms a good safety profile, with the only concern of genital infections. Concerns on euglycemic diabetic ketoacidosis, hypoglycemia in the elderly, urinary infections, bone fractures, use in patients with impaired kidney function, vanished, in the absence of an even minimal signal of danger. That is a striking difference when we refer to the alarms reported by FDA and EMA, and it will require a cautious clarification through future studies.

To conclude, several arguments can be provided either in favor of an enthusiastic or a more cautious interpretation of the *Empa*-*Reg outcome* results: it is too early to pretend answering to all the points raised by the study. Trying a parallelism, similarly to how intensive vs conventional treatment strategy trials had imposed a radical change in the therapeutic approach, *Empa*-*Reg outcome* challenges us, forcing to change again the clinical paradigm followed in the last years. To be provocative, we might figure out to go back to more rigid and simple therapeutic schemes, with a potentially new hierarchy of drug choice to combine with metformin at least in patients with high CV risk.
